# Improving Nursing Home Readiness: PPE Access and Use During COVID-19

**DOI:** 10.1093/geroni/igaf122.3965

**Published:** 2025-12-31

**Authors:** Melissa Taylor, Anna Jones, Steven Miller, Linda Anderson, Lori Popejoy, Amy Vogelsmeier

**Affiliations:** University of Missouri - Columbia, Columbia, Missouri, United States; University of Missouri - Columbia, Columbia, Missouri, United States; University of Missouri - Columbia, Columbia, Missouri, United States; University of Missouri - Columbia, Columbia, Missouri, United States; University of Missouri - Columbia, Columbia, Missouri, United States; University of Missouri - Columbia, Columbia, Missouri, United States

## Abstract

The COVID-19 pandemic created unprecedented challenges for nursing homes (NHs), particularly challenges related to personal protective equipment (PPE). This analysis explored PPE access and use by NH staff, residents, and visitors during the pandemic. As part of a larger study aimed at developing recommendations to improve NHs’ preparedness for infectious disease outbreaks, 116 interviews were conducted with leaders (n = 43), staff (n = 140), infection preventionists (n = 16) and residents/families (n = 63) from 24 Missouri NHs. Data were analyzed using directed content analysis to examine patterns of PPE access, usage, and challenges. To further verify challenges related to PPE access and use during the pandemic, a systematic review of 31 articles published between 2020 and 2023 was conducted. Interview findings revealed that all 24 NHs experienced PPE shortages due to supply chain disruptions and had to rely on corporate, state, and community support. Despite federal mandates, PPE use was inconsistent due to supply shortages, staff resistance, and communication difficulties with residents with dementia and hearing impairments. Training staff on PPE use and adapting care during shortages were key concerns. The systematic review findings echoed the interview findings, revealing a lack of streamlined PPE acquisition systems for NHs, significantly affecting supply and use. Few innovative solutions for maintaining resident care during PPE shortages were documented. Recommendations include ensuring NHs have reliable access to PPE, awareness of supply resources, and inclusive implementation processes. Special consideration for PPE use should be given to residents with memory and/or hearing impairment challenges.

